# Urinary Kininogen-1 and Retinol binding protein-4 respond to Acute Kidney Injury: predictors of patient prognosis?

**DOI:** 10.1038/srep19667

**Published:** 2016-01-21

**Authors:** Laura Gonzalez-Calero, Marta Martin-Lorenzo, Angeles Ramos-Barron, Jorge Ruiz-Criado, Aroa S. Maroto, Alberto Ortiz, Carlos Gomez-Alamillo, Manuel Arias, Fernando Vivanco, Gloria Alvarez-Llamas

**Affiliations:** 1 Department of Immunology, IIS-Fundacion Jimenez Diaz-UAM, REDinREN, Madrid, Spain; 2 Nephrology Department, Valdecilla Hospital, University of Cantabria, Instituto de Formación e Investigación Marqués de Valdecilla, IDIVAL, Santander, Cantabria, Spain; 3 Department of Nephrology/IRSIN, IIS-Fundación Jiménez Díaz, School of Medicine, UAM, Madrid, Spain; 4 Department of Biochemistry and Molecular Biology I, Universidad Complutense, Madrid, Spain

## Abstract

Implementation of therapy for acute kidney injury (AKI) depends on successful prediction of individual patient prognosis. Clinical markers as serum creatinine (sCr) have limitations in sensitivity and early response. The aim of the study was to identify novel molecules in urine which show altered levels in response to AKI and investigate their value as predictors of recovery. Changes in the urinary proteome were here investigated in a cohort of 88 subjects (55 AKI patients and 33 healthy donors) grouped in discovery and validation independent cohorts. Patients’ urine was collected at three time points: within the first 48 h after diagnosis(T1), at 7 days of follow-up(T2) and at discharge of Nephrology(T3). Differential gel electrophoresis was performed and data were confirmed by Western blot (WB), liquid chromatography/mass spectrometry (LC-MS/MS) and enzyme-linked immunosorbent assay (ELISA). Retinol binding protein 4 (RBP4) and kininogen-1 (KNG1) were found significantly altered following AKI. RBP4 increased at T1, and progressively decreased towards normalization. Maintained decrease was observed for KNG1 from T1. Individual patient response along time revealed RBP4 responds to recovery earlier than sCr. In conclusion, KNG1 and RBP4 respond to AKI. By monitoring RBP4, patient’s recovery can be anticipated pointing to a role of RBP4 in prognosis evaluation.

Acute kidney injury (AKI) is a multifactorial syndrome, a frequent clinical complication in hospitalized patients and responsible of a substantial morbidity and mortality^1^,^2^. Serum creatinine (sCr) is the reference standard for AKI diagnosis, but lacks sensitivity, may be delayed in response to injury (retrospective indicator), and is influenced by independent factors. As result, current diagnosis is often delayed in time, limiting the possibility of earlier intervention and promoting adverse outcomes. AKI may progress to chronic kidney disease (CKD), CKD itself is a risk factor for AKI in CKD patients, and AKI may accelerate CKD progression. In fact a substantial proportion of AKI patients in daily clinical practice have underlying CKD. In this context, design of novel therapeutic strategies aimed at limiting kidney injury and preventing AKI progression requires novel biomarkers that allow early monitoring of kidney damage and helping in prognosis prediction^3^,^4^.

Urine represents a combination of both plasma ultrafiltrate and urinary tract proteins, including glomerular filtrated plasma proteins, soluble proteins secreted by tubular epithelial cells and microvesicles as exosomes. Thus, urine composition not only reflects normal kidney function but also contains specific kidney produced proteins which may be altered in response to underlying physiopathology^5^. Urine has been investigated to study renal physiology and kidney diseases^6^. Our group have investigated urine in the search for novel molecular targets of potential use in the clinical setting from a diagnosis or prognosis point of view in the context of diabetic nephropathy^7^, chronic kidney disease^8^, and cardiovascular disease^9^. Here, we pursued the identification of major molecular alterations in urine in response to AKI in the search for novel indicators. Their capability to monitor recovery from AKI and thus predict patient’s prognosis will be also evaluated.

## Results

### Urinary RBP4 and KNG1 proteins are identified as main responders to AKI

**Figure 1:**
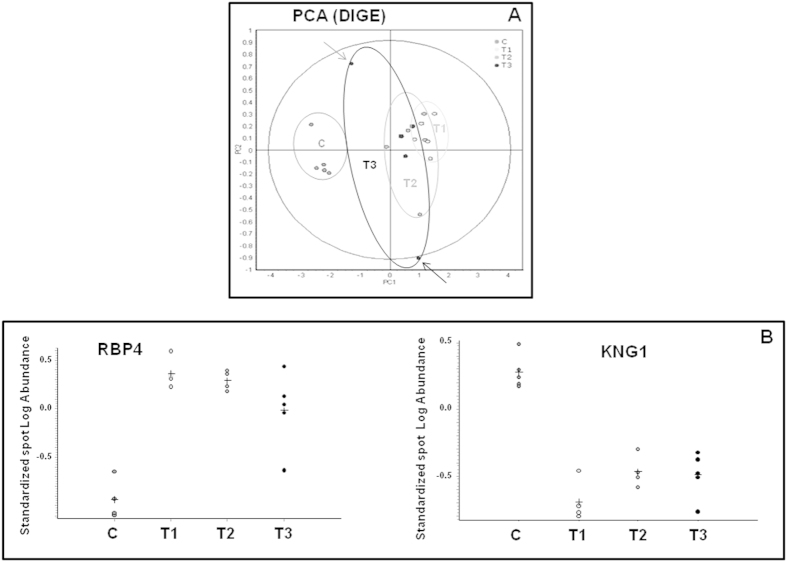
Proteins significantly altered in urine in response to AKI. (**A**) Principal component analysis (PCA) graph resulting from the DIGE analysis show clear grouping of control subjects and AKI patients. Each dot represents a pool sample made of urine aliquots from 4 individuals. Urine was collected from a total of 20 healthy subjects (C) and 20 patients at three different time points (T1, T2 and T3). Arrows point to individuals showing trend of partial recovery. (**B**) Standardized Log Abundance found corresponding to protein spots of RBP4 and KNG1. A clear increase in response to AKI was observed for RBP4 and the opposite trend was found for KNG1.

Table 1 depicts demographic and clinical characteristics of participants. Individual data are included in Supplementary Tables. Principal component analysis (Fig. 1A) revealed a clear differentiation between controls and cases with a slight trend towards recovery gradually over time for a sub-group of individuals. Differential statistical analysis revealed Retinol binding protein 4 (RBP4) and Kininogen 1 (KNG1) as main molecular responders to AKI condition (Fig. 1B and Table 2). RBP4 is increased significantly in the first sample (T1) compared to control values, and remains significantly higher at T2. Inter-individual variability was particularly observed at nephrological discharge (T3). The opposite trend was observed for KNG1, decreasing its urinary concentration in response to AKI from the first time point evaluated (T1). At discharge, KNG1 urinary levels remain considerably lower than control values.

**Figure 2:**
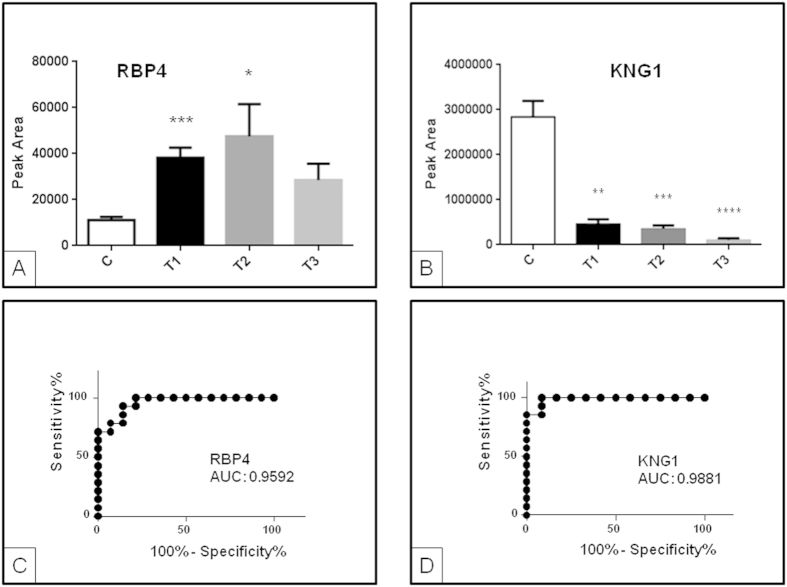
Urinary RBP4 (**A**) and KNG1 (**B**) analysis by (SRM)LC-MS/MS. Transitions shown (precursor→fragment) are: 813.48->970.60 (RBP4) and 713.70->956.0 (KNG1). A different cohort of individuals to that used in DIGE analysis was recruited. Increased urinary levels for RBP4 in response to AKI were confirmed. Error bars show standard error of media. ROC curves showing response to AKI were calculated for RBP4 (**C**) and KNG1 (**D**). *p value ≤ 0.05; **p value ≤ 0.01; ***p value ≤ 0.001; ****p value ≤ 0.0001.

In the confirmation cohort, target analysis of urinary RBP4 and KNG1 was performed by liquid chromatography mass spectrometry in selected reaction monitoring mode (SRM-LC-MS/MS) for validation. In this case, individual urine samples were analyzed to assess individual behavior (molecular response). Again, significant differences were observed between control group and AKI patients for RBP4 and KNG1 (Fig. 2) in the first 48 h and follow-up, confirming previously observed trends by differential gel electrophoresis (DIGE) and the response of these two proteins (RBP4 increase and KNG1 decrease) to AKI. This response was further evaluated by ROC curves, resulting in areas under the curve (AUC) higher than 0.9 in both cases (see Fig. 2). While average KNG1 levels do not return to control values at discharge, there is a clear trend towards normalization in the case of RBP4 (Fig. 2).

### RBP4 in urine responds to patient’s recovery earlier than sCr clinical marker

**Figure 3:**
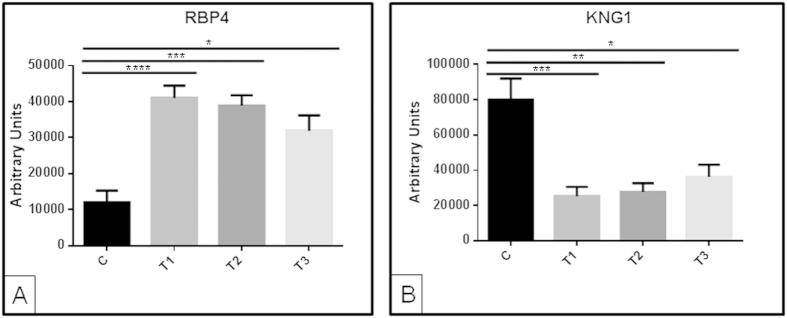
Western blot data for RBP4 performed with individual urine samples. A different cohort of individuals to that used in DIGE analyses was recruited. On average, increased urinary levels for RBP4 (**A**) and decreased urinary levels for KNG1 (**B**) in response to AKI was confirmed: graphs show average data for all patients monitored (recovered at any time point or not). *p value ≤ 0.05; **p value ≤ 0.01; ***p value ≤ 0.001; ****p value ≤ 0.0001.

To further investigate a potential prognostic value for RBP4, we analyzed individual samples collected from a new randomized cohort of a total of 23 patients and 13 healthy subjects by WB. Thirteen of those 23 patients could be monitored at the three time points. On average, including all patients monitored (and not only those for whom sampling was possible at the three time points), RBP4 increased significantly at T1 and progressively decreased thereafter towards normalization, as expected in view of DIGE and SRM data (Fig. 3A). Persistent decrease in KNG1 was observed from T1 (Fig. 3B).

**Figure 4:**
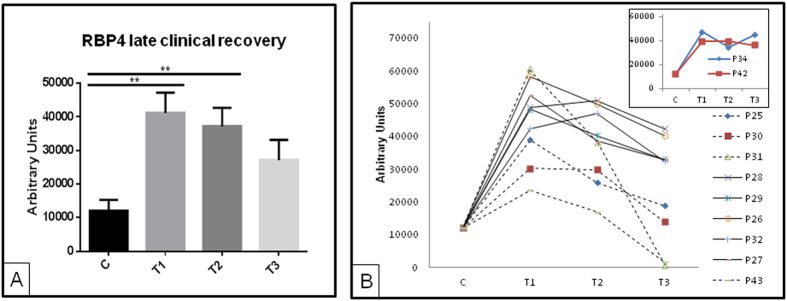
WB data for RBP4 and KNG1 for patients who clinically recovered later than T3 according to sCr. Normalization of RPB4 levels was observed already at T3 on average (**A**,**B**) Individual RBP4 responses for those patients monitored at the three time points (solid lines represent patients with sCr(T3) > 1.3 mg/dL and dashed lines represent patients with sCr(T3) ≤ 1.3 mg/dL). Inset shows representative behavior of RBP4 for patients who do not recover. **p value ≤ 0.01.

In a further step, patients’ response was analyzed individually in view of their recovery. In daily clinical practice a substantial proportion of AKI patients have underlying CKD (30% in our series) as does a significant proportion of the general population (around 10%)^10^. Thus, inclusion of CKD patients, as in this study, represents the AKI patients clinicians are dealing with^11^. Lack of clinical recovery means that CKD may have developed (if there was no prior CKD) or that CKD has progressed (in patients with prior CKD). Both outcomes are associated with impaired vital outcomes, and early prediction of *de novo* or worsening CKD may be equally useful to identify a high risk population that may be enrolled in future clinical trials. In this study, recovery means sCr values ≤1.3 mg/dL by T3 for non-CKD patients of returning to basal levels for CKD individuals, according to clinical criteria. Figure 4A represents those patients who clinically recover later than T3 according to sCr values, showing normalized RPB4 values (no significant differences between C and T3) already at T3. Individual response of urinary RBP4 also shows a decreasing trend towards control values after AKI for those patients who recover (Fig. 4B). Patients with sCr(T3) ≤ 1.3 mg/dL already showed RBP4 values close to controls at this time point (Fig. 4B, dashed lines), while in patients who do not recover urinary RBP4 is persistently high (Fig. 4B, insert). These results were further confirmed by ELISA as can be seen in Supplementary Figure 3. RBP4 is, thus, able to show patient’s recovery earlier than sCr.

## Discussion

The human urinary proteome was here first investigated in the search for significantly altered molecules in response to AKI^12^. Prior studies point to the need of defining marker panels preferably than individual diagnostic molecules to better assess the complex AKI/CKD syndromes^13^. Proteomics approaches provide non-biased information on the protein changes in a biological fluid as a whole, without pre-selection of potential targets of interest. DIGE technology was chosen here for the discovery phase, based on its proved value as a differential proteomics strategy for proteins quantification in biological samples in the search for markers of disease (cardiovascular disease, kidney disease, cancer and neurodegenerative diseases, among others), by our group and others^3^,^14^,^15^,^16^,^17^,^18^,^19^. KNG1 and RBP4 were found here as responders to AKI from the first sample obtained within 48 h of nephrological diagnosis. These data were confirmed in an additional cohort of individuals and prompted the development of a specific mass spectrometry-based quantitative method specifically developed for high-throughput assessment of KNG1 and RBP4 proteins in urine, in a manner which may offer a future reliable quantitative assay which may be competitive in the clinical setting. Not only earlier diagnosis but also outcome prediction after an AKI episode is of enormous interest. Insulin-like growth factor-binding protein 7 (IGFBP-7) was recently identified in urine as a prognostic marker^3^ and, here, the capacity to anticipate patient’s recovery earlier than traditional markers as sCr was further evaluated and confirmed for RBP4.

KNG1and RBP4 genes were two of the top kidney up-regulated ones in an end stage renal disease (ESRD) mouse model^20^. We show here a significant decrease in urinary KNG1 in response to AKI. Kininogen is a precursor of kinins from the kallikrein-kinin system. When this cascade is activated, the serine protease kallikrein (tissue or plasma) processes kininogen (low or high molecular weight) to produce vasoactive peptides (e.g. bradykinin (BK)) which are involved in blood pressure regulation, renal and cardiac function and inflammation among other physiological and pathological processes^21^,^22^. In a salt-induced hypertension rat model, tissue kallikrein/kinin infusion reversed kidney injury, inflammation and fibrosis^23^ pointing to a protective role for this metabolic cascade^24^,^25^. In hypertensive subjects, urinary kininogen and kinins were decreased, while excretion of kallikrein was normal. However, in end-stage renal disease, kinins and kallikrein were decreased and a normal excretion of kininogen was found^26^. In IgA nephropathy patients, urinary kininogen excretion was found significantly different for responders and non-responders to an angiotensin-converting-enzyme inhibitor (ACE-inhibitor) therapy^27^, which may be in alignment with the reported variants of kininogen gene influencing individual response to aldosterone^28^. To our best knowledge, urinary KNG1 has not been directly related to AKI in humans. T-Kininogen was found differentially expressed in rats after cisplatin or ischemia/reperfusion treatment^29^. In response to streptozotocin-induced diabetes in rats, kininogen, kallikrein, BK levels and BK B1- and B2-receptor expression were decreased in glomeruli and in cultured podocytes, implying a role for kallikrein-kinin system in podocyte apoptosis in diabetic conditions^30^. Plasma high molecular weight kininogen increased in accordance to progressive renal function worsening in microalbuminuric diabetic patients^31^, the opposite trend to what previously reported in urine. However, plasma kininogen was decreased in biopsy confirmed acute rejection after renal transplantation^32^, following the same trend that urine kininogen in response to kidney chronic allograft dysfunction^33^. These two studies are in consonance with our findings. Despite these studies, the participation and specific role of the molecules constituting the kallikrein-kinin system in a wide variety of clinical contexts are underexplored. Nevertheless, there are enough evidences to propose a link between kininogen expression and kidney injury (e.g. AKI, rejection following transplantation), loss of renal function, hypertension or albuminuria progression.

We also found increased urinary levels of RBP4 in response to AKI. The rise was observed from the first 48 h of nephrological diagnosis and maintained at discharge on average, although individual differences were further explored and confirmed related to recovery. This low molecular weight protein is easily filtered by the glomerulus and reabsorbed for the most part in proximal tubules. It has been proposed as the best indicator of tubular dysfunction, as a minor reduction in tubular function may lead to increased RBP4 excretion. In consequence, urinary RBP4 level has been proposed as the most sensitive biomarker for loss of proximal renal tubule function in humans^34^. In response to acute kidney allograft injury, RBP levels increased in urine^35^. In a follow-up study of kidney transplantation prognosis in the following year, RBP predicted graft loss independently of histology and urinary albumin^36^. Increased levels of RBP4 in diabetic patients with further increased observed in presence of microalbuminuria pointed to an incipient nephropathy in this clinical group^37^. In this context, RBP4 has been proposed as a marker of risk of developing CKD of macroalbuminuric diabetic nephropathy patients^38^.Other studies observed altered RBP4 levels in diabetic patients with tubular injury, although it is unclear whether it predicts microalbuminuria development^39^,^40^,^41^. Related to AKI, there is increasing interest in further investigating the role of RBP4 in early diagnosis. Data shown here give further evidence of the RBP4 (reversible) response to the AKI clinical condition, showing increased urinary RBP4 levels in AKI patients. Furthermore, RBP4 response in urine tends to normalize with time for those patients who progressively recover in terms of sCr, however RBP4 remains persistently high in those patients who do not recover. More importantly, RBP4 responds earlier than sCr does on average and allows monitoring individual response and further predict future recovery.

The coexistence of common mechanisms in different renal disorders with subjacent complex cellular responses taking place simultaneously make the specific identification of biomarkers of AKI, allograft dysfunction, diabetic renal complications or CKD development a real challenge. Some of the proposed biomarkers in the literature are in fact coincident in various diseases^42^.

The use of panels of biomarkers to the detriment of individual molecules should be explored to improve accuracy and specificity. A specific assay for these RBP4 and KNG1 proteins quantitation, based on SRM methodology, was here developed which enormously facilitates feasibility in the clinic in terms of sensitivity and throughput.

One limitation of the study may be the number of recruited subjects, wide distribution for age, diabetes, hypertension or drugs. On the other hand, serum creatinine (sCr) and urinary output are the key clinical tools to define AKI. However, assessment of urinary output is more prone to error, especially in patients without an indwelling bladder catheter^43^. Thus and in order to reflect daily clinical situation in many non-intensive care units (ICU), we relied on serum creatinine criteria for diagnosis of AKI. DIGE also has known weaknesses to be acknowledged, such as poor coverage of low abundance proteins or very hydrophobic proteins. On the other hand, urine has enormous potential as non-invasive fluid able to reflect changes in renal function or injury, but the variability in protein content may be considerable and this fact implies normalization to e.g. urinary creatinine or total protein content. Finally, real clinical translation of a novel identified marker is so far hampered by significant difficulties. In general, the biomarker pipeline from identification to final implementation should go through several essential process phases: candidate discovery, qualification, verification, research assay optimization, biomarker validation, commercialization and widespread implementation in routine clinical practice^44^,^45^. Currently, no novel biomarker has progressed to all those stages in AKI. In particular, AKI occurs in a wide variety of clinical scenarios, thus assessing the real-life performance of a proposed marker implies prior testing of its sensitivity/specificity and capacity of prediction in these various clinical conditions^46^.

## Conclusions

Urinary KNG1 and RBP4 clearly respond to AKI. Additionally, RBP4 could predict recovery by monitoring this protein levels over time after AKI, as RBP4 reflects patient’s normalization earlier than sCr values do. Further evaluation of KNG1 and RBP4 diagnostic and prognostic value should follow (e.g. in a prospective study where their role in earlier diagnosis could be investigated), alone or in combination with other proposed markers.

## Methods

### Subjects recruitment and urine samples collection

Urine samples from 55 AKI patients without proteinuria, diagnosed by Nephrology Department staff, were collected at Hospital Valdecilla (Santander, Spain) at three time points: diagnosis within the first 48 h (T1), at 7 days of follow-up (T2) and at Nephrology discharge (T3). AKI was defined and classified according KDIGO criteria^47^. Peak sCr was considered as the highest value of sCr after diagnosis. Recovery was considered, as suggested by ADQI guidelines, when sCr was ≤1.3 mg/dL in patients without previous CKD or return to baseline values in case of CKD. Patients were ramdomized and samples from 20 patients were used in a first discovery phase, and samples from 35 patients were recruited for confirmation studies. Urine samples were also collected from 33 healthy donors (mean age 47.9 ± 12 (26–65), without comorbidities and normal renal function) (20 for the discovery phase and 13 for confirmation). Existence of cardiovascular disease (CVD), CKD, hypertension (HTN) or diabetes mellitus was assessed. Spot urine samples were obtained from a morning random-catch sample, collected in a sterile container, centrifuged to remove cell debris (3000 rpm, 10 min) (Centrifuge Sorvall T6000D), and the supernatant frozen at −80 °C until analysis.

### Ethics, consent and permissions

Sample collection procedures were in accordance with the Helsinki declaration and signed informed consent was obtained. Approved consent was obtained from Ethical Committee for Clinical Research from Cantabria (CEIC, Comité de Ética e Investigación Clínica), endorsed by IDIVAL.

### Unbiased proteomics analysis by Differential Gel Electrophoresis (DIGE)

Differential protein analysis was performed by DIGE (GE Healthcare®). A total of 20 AKI patients and 20 control subjects were used in this first discovery phase, to identify strongest protein alterations in urine in response to AKI. Four individual urine samples from each group were pooled, making a total of 5 pools to be analysed per group (C, T1, T2 and T3). 5 nmol CyDye DIGE Fluor labelling kit (GE Healthcare) was used to label proteins following manufacturer’s instructions. Labelled samples were loaded onto IPG strips (24 cm, pH 4–7) and isoelectric focusing (IEF) was carried out in a PROTEAN IEF CELL (BioRad)^14^. Second dimension was carried out on 14% running gels using EttanDaltSix System (GE Healthcare). Gels were scanned using a Typhoon 9400 Variable Mode Imager (GE Healthcare) and spot maps were processed, analyzed and compared using the DeCyder Differential Analysis Software version 6.5 (GE Healthcare). Spot detection and normalized volume ratio calculations were performed in the Differential In-gel Analysis (DIA) module, while gel-to-gel matching and statistical analysis were performed in the Biological Variation Analysis (BVA) module. A first ANOVA test was performed (including FDR) followed by Student’s t test for pairs comparisons of the expression data of each spot.

### MALDI-TOF Mass Spectrometry identification of candidate markers of AKI

Spots selected for analysis were in-gel reduced, alkylated and digested with trypsin according to Sechi and Chait^48^. Supernatants were spotted onto a matrix assisted laser desorption ionization (MALDI) target plate. 0.5 μl of a 3 mg/ml of α-cyano-4-hydroxy-cinnamic acid matrix in 0.1% trifluoroacetic acid-50% acetonitrile was added to the dried peptide digest spots. Samples were analyzed using MALDI-time of flight (TOF/TOF) mass spectrometer 4800 plus Proteomics Analyzer (Applied Biosystems. MDS Sciex, Toronto, Canada) and 4000 Series Explorer™v 3.5 Software (ABSciex). GPS explorer v 3.5 (ABSciex) software was used for spectra analyses and generating peaking lists. Searches for peptide mass fingerprints, tandem MS spectra and both combined were performed in the NCBInr database using Mascot v.2.2 from Matrix Science (http://www.matrixscience.com). In all identified proteins, the probability score was greater than the one fixed by Mascot as being significant, that is, a p value under 0.05.

### Urinary RBP4 and KNG1 targeted analysis by Selected Reaction Monitoring (SRM) LC-MS/MS

As previously published by our group and others^7^,^9^,^49^, we used SRM-LC-MS/MS to validate differential proteins identified in the discovery phase. Urine samples from different individuals’ cohorts were used. In particular, 16 patients and 7 healthy subjects were recruited. Urine samples were concentrated and desalted. Total protein content was quantified by Bradford assay and protein samples were reduced, alkylated and digested with sequencing grade trypsin (Roche). Tryptic peptides solutions were cleaned with C18 spin columns (Protea Biosciences) according to manufacturer’s instructions and mixed 1:1 with mobile phase A (0.1% formic acid in MilliQ water). A 6460 Triple Quadrupole mass spectrometer was used on-line connected to nano-chromatography in a Chip-format configuration (ChipCube interface, ProtID Zorbax 300B-C18-5 μm chip, 43 × 0.075-mm analytical column and 40 nL enrichment column, Agilent Technologies). The system was controlled by Mass Hunter Software (v4.0 Agilent Technologies). Theoretical SRM transitions were designed using Skyline (v.1.1.0.2905) and peptide specificity was confirmed by protein blast. Only those proteotypic (specific) peptides were selected^50^. In this case, one specific proteotypic peptide was measured per protein, by monitoring two transitions for RBP4 and three transitions for KNG1 (see Supplementary material).

### Urinary RBP4 and KNG1 assessment by Western blot and ELISA

A different cohort to that used in the discovery phase was recruited for confirmation of candidate markers by WB. 10 mL urine samples were collected from 23 AKI patients and 13 healthy individuals. The same amount of total protein (10 μg) total protein was dissolved in Laemmli buffer and loaded per lane in a 12% acrylamide gel (4% stacking). Membranes were blocked with PBS containing 7.5% non-fat dry milk powder and 0.1% Tween-20 for 1 h at room temperature. Membranes were then incubated for 1 h with specific primary antibody rabbit monoclonal anti-KNG1 (1:5000) (Abcam) or with rabbit monoclonal anti-RBP4 (1:50000) (Abcam) in phosphate buffered saline in Tween-20 (PBS-T) (0.05%) containing 5% non-fat dry milk. Finally, they were incubated with horseradish peroxidase (HRP)-conjugated rabbit TrueBlot® (1:1000) (Rockland) for KNG1 or HRP-conjugated goat anti-rabbit (1:10000) (Nordic) for RBP4 as secondary antibody, in PBS-T (0.05%) containing 5% non-fat dry milk. Detection was performed by enhanced chemiluminescence (ECL kit; GE Healthcare) following the instructions of the manufacturer. Protein bands were quantified by ImageJ software.

Concentration of RBP4 was additionally measured by ELISA in urine from a cohort of 8 control subjects and 8 AKI patients following manufacturer’s instructions (Abcam, ab108897).

### Statistical analysis

Statistical analyses were performed using SPSS, v15.0 (SPSS Inc., Chicago, IL, USA), and GraphPad Prism 6 (version 6.01) software. The Kolmogorov-Smirnov test (KS) was used to assess the normal distribution of clinical variables. Quantitative variables were expressed as mean ± standard deviation (SD) or median and interquartile range (IR) for variables with non-normal distribution. Kruskal-Wallis with Dunn’s multiple comparisons test was calculated and differences were considered significant when p value < 0.05. For receiver operating characteristic (ROC) curve, assessment confidence level was selected as 95% and p value < 0.0001.

## Additional Information

**How to cite this article**: Gonzalez-Calero, L. *et al.* Urinary Kininogen-1 and Retinol binding protein-4 respond to Acute Kidney Injury: predictors of patient prognosis? *Sci. Rep.* **6**, 19667; doi: 10.1038/srep19667 (2016).

## Supplementary Material

Supplementary Information

## Figures and Tables

**Table 1:** Demographics characteristics of patients included in the study.

Characteristics	N (%)
Age, years	64 ± 16† (15–84)
Men	37 (67.3)
Comorbidities	
Hypertension	41 (74.5)
Diabetes mellitus	21 (38.2)
Cardiovascular disease	28 (50.9)
Chronic kidney disease	16 (29.1)
Charlson comorbidity score	5.6 ± 3.4†
ISI comorbidity score	0.28 ± 0.19†
Nephrotoxic drugs (≥1)	47 (85.5)
Type of admission	
Medical	39 (70.9)
Surgical	16 (29.1)
ICU stage	12 (21.8)
Etiology of acute kidney disease	
Pre-renal	28 (50.9)
Acute tubular necrosis	27 (49.1)
AKI–KDIGO at diagnosis	
1	5 (9.1)
2	9 (16.4)
3	41 (74.5)
Peak sCr	8.1 ± 4.7†
Length of stay, days	9 (8)*
Dialysis required	15 (27.3)
Oliguria (≤400 mL/24 h)	10 (20)
Full recovery of renal function at discharge	17 (34.7)
Hospital mortality	3 (5.5)
Mortality at one year	7 (12.7)

^*^Expressed as median and interquartile range.

^†^Expressed as mean ± SD.

**Table 2:** T-Test and average data values for RBP4 and KNG1 protein spots as main significantly varied proteins over time in response to AKI.

Protein	T1/C		T2/C		T3/C		1-ANOVA
	T-test	Av.Ratio	T-test	Av.Ratio	T-test	Av.Ratio	
**KNG1**	0.001	−9.13	0.009	−5.57	0.07	−5.76	0.0004
**RBP4**	0.0008	19.06	0.009	15.69	0.1	9.93	0.0008
