# Supplementary material for: Urinary Kininogen-1 and Retinol binding protein-4 respond to Acute Kidney Injury: predictors of patient prognosis?
Source: Sci Rep. 2016 Jan 21;6:19667. doi: 10.1038/srep19667 (PMC4726181; doi:10.1038/srep19667)
Supplement: Supplementary Information [file srep19667-s1.pdf]

Urinary Kininogen-1 and Retinol binding protein-4 respond to Acute Kidney Injury: predictors of patient prognosis?

Laura Gonzalez-Calero[1], Marta Martin-Lorenzo[1], Angeles Ramos-Barron[2], Jorge Ruiz-Criado[2], Aroa S. Maroto[1], Alberto Ortiz[3], Carlos Gomez-Alamillo[2], Manuel Arias[2], Fernando Vivanco[1,4], Gloria Alvarez-Llamas[1*]

[1] Department of Immunology, IIS-Fundacion Jimenez Diaz-UAM, REDinREN, Madrid, Spain.
[2] Nephrology Department, Valdecilla Hospital, University of Cantabria, Instituto de Formación e Investigación Marqués de Valdecilla, IDIVAL, Santander, Cantabria, Spain.
[3] Department of Nephrology/IRSIN, IIS-Fundación Jiménez Díaz, School of Medicine, UAM, Madrid, Spain.
[4] Department of Biochemistry and Molecular Biology I, Universidad Complutense, Madrid, Spain.

*To whom correspondence should be addressed: Gloria Alvarez-Llamas, PhD. IIS-Fundacion Jimenez Diaz. Avenida Reyes Catolicos 2, 28040 Madrid (Spain). Phone N. +34 915504800. Fax N. +34 915448246. Email: galvarez@fjd.es

Email addresses: laura.gcalero@fjd.es; marta.martin@fjd.es; ramosbarron@gmail.com; asanzmaroto@gmail.com; aortiz@fjd.es; cgalamillo@gmail.com; nefarm@gmail.com; fvivanco@fjd.es; galvarez@fjd.es

Supplementary Table 1. Clinical data of AKI patients. Urine samples were collected within the first 48h (1, Cr1), follow-up (2, Cr2) and at discharge (3, Cr3). For each patient, the approach (study) and time point (T) in which the urine sample was used is detailed: DIGE (discovery phase), WB (confirmation phase), SRM (confirmation phase) or ELISA (confirmation phase). Cr0: baseline serum creatinine, HTN: hypertension, CRF: chronic renal failure, CVD: cardiovascular disease, Cr: serum creatinine.

Patient N.	*Analysis approach (T)*	*HTN*	*Diabetes*	*CKD*	*CVD*	*Cr1 (mg/dL)*	*Cr2 (mg/dL)*	*Cr3 (mg/dL)*
P1	DIGE (1,2,3)	Yes	Yes	No	Yes	3.1	1.2	0.9
P2	DIGE (1,2,3)	Yes	No	No	No	9.1	1.6	1.2
P3	DIGE (1,2,3)	Yes	Yes	No	No	5.2	3.7	1.2
P4	DIGE (1,2,3)	Yes	No	Yes	No	7.9	3.4	2.2
P5	DIGE (1,2,3)	Yes	No	No	Yes	3.4	2.9	1.7
P6	DIGE (1,2,3)	No	No	No	No	8.0	7.5	6.4
P7	DIGE (1,2,3) ELISA (1,2,3)	No	No	No	Yes	4.8	2.0	1.5
P8	DIGE (1,2,3)	Yes	No	No	Yes	1.5	1.1	1.0
P9	DIGE (1,2,3) ELISA (1,2,3)	Yes	Yes	No	Yes	8.1	7.8	1.2
P10	DIGE (1,2,3)	No	No	No	No	2.2	2.0	1.5
P11	DIGE (1,2,3) ELISA (1,2,3)	Yes	No	No	Yes	9.7	7.7	5.0
P12	DIGE (1,2,3)	Yes	No	No	No	6.2	7.3	6.5
P13	DIGE (1,2,3)	No	No	No	No	2.0	1.2	0.9
P14	DIGE (1,2,3)	Yes	No	No	Yes	3.1	2.7	1.7
P15	DIGE (1,2,3)	Yes	Yes	No	Yes	2.0	1.8	-
P16	DIGE (1,2,3)	Yes	Yes	No	No	9.8	7.1	0.9
P17	DIGE (1,2,3)	Yes	Yes	No	No	1.6	-	1.0
P18	DIGE (1,2,3)	Yes	Yes	No	Yes	12.5	9.2	4.3
P19	DIGE (1,2,3)	No	No	No	No	9.0	3.1	1.5

P20	DIGE (1,2,3)	Yes	Yes	Yes	Yes	3.2	2.8	2.3
P21	WB (1)	Yes	Yes	Yes	No	4.3	3.2	2.5
P22	WB (1)	Yes	No	No	No	6.5	2.7	1.2
P23	WB (1)	Yes	No	No	No	1.2	1.5	-
P24	WB (1,2)	Yes	Yes	Yes	Yes	2.5	3.0	4.2
P25	WB (1,2,3) ELISA (1,2,3)	No	Yes	No	Yes	4.8	1.4	0.9
P26	WB (1,2,3)	Yes	Yes	No	Yes	3.8	2.8	2.2
P27	WB (1,2,3) ELISA (1,2,3)	Yes	No	No	No	4.2	2.5	1.9
P28	WB (1,2,3)	Yes	Yes	No	Yes	2.3	2.4	2.1
P29	WB (1,2,3)	No	No	No	No	9.1	15.6	8.0
P30	WB (1,2,3)	No	No	No	No	3.9	2.0	1.2
P31	WB (1,2,3) ELISA (1,2,3)	Yes	Yes	No	Yes	9.2	1.2	0.9
P32	WB (1,2,3)	No	No	No	No	3.8	5.5	1.7
P33	WB (1,2,3) ELISA (1,2,3)	Yes	No	No	Yes	4.4	4.3	3.9
P34	WB (2)	Yes	No	Yes	Yes	7.4	5.8	4.6
P35	WB (2)	Yes	No	No	Yes	10.0	9.8	5.9
P36	WB (2,3)	Yes	No	Yes	Yes	10.1	4.5	3.4
P37	WB (2,3)	No	No	Yes	Yes	4.4	5.1	4.7
P38	WB (2,3)	Yes	Yes	Yes	Yes	5.8	6.8	3.7
P39	WB (3)	Yes	Yes	Yes	No	13.8	10.6	9.5
P40	WB (1,2,3) SRM (3)	Yes	Yes	No	Yes	4.1	2.0	1.6
P41	WB (1,2,3) SRM (3)	Yes	No	No	No	3.5	2.7	1.0
P42	WB (1,2,3) SRM (3) ELISA (1,2,3)	Yes	No	Yes	No	23.4	10.3	10.0
P43	WB (1,2,3) SRM (1,2)	Yes	No	No	No	5.6	2.5	1.3
P44	SRM (1)	No	No	Yes	Yes	6.5	1.6	5.7
P45	SRM (1)	Yes	Yes	Yes	Yes	2.1	-	1.4
P46	SRM (1)	Yes	No	Yes	Yes	8.0	4.1	3.0
P47	SRM (1,2)	Yes	Yes	No	Yes	4.3	2.0	1.4

P48	SRM (1,2)	No	No	Yes	No	6.1	7.5	8.9
P49	SRM (1,2)	Yes	No	No	No	5.1	2.7	1.0
P50	SRM (2)	Yes	No	No	No	6.9	1.6	-
P51	SRM (2)	Yes	No	No	No	2.0	0.8	0.6
P52	SRM (2)	No	No	No	No	2.0	1.5	1.1
P53	SRM (3)	Yes	Yes	Yes	Yes	2.8	1.7	1.8
P54	SRM (3)	Yes	Yes	Yes	Yes	4.8	4.3	3.3
P55	SRM (3)	No	No	No	No	17.8	18.6	16.6

Supplementary Table 2. Control subjects and study in which they have been included.

Control N.	*Analysis approach*	*Age*	*Sex*
Discovery Phase			
1	DIGE	58	F
2	DIGE	36	F
3	DIGE	52	M
4	DIGE	26	F
5	DIGE	58	F
6	DIGE	48	F
7	DIGE	27	F
8	DIGE, ELISA	39	M
9	DIGE	59	F
10	DIGE, ELISA	55	F
11	DIGE, ELISA	27	F
12	DIGE	40	M
13	DIGE, ELISA	63	F
14	DIGE	26	F
15	DIGE	45	F
16	DIGE	56	M
17	DIGE	59	F
18	DIGE	31	F
19	DIGE	55	M
20	DIGE	50	F
Confirmation Phase			
21	WB, SRM	59	M
22	WB, SRM, ELISA	60	F
23	WB, SRM, ELISA	50	F
24	WB, SRM	60	M
25	WB, SRM, ELISA	65	M
26	WB, SRM	40	M
27	WB, ELISA	50	F
28	WB	56	M
29	WB	54	F
30	WB, SRM	48	F
31	WB	58	F
32	WB	37	F
33	WB	33	F

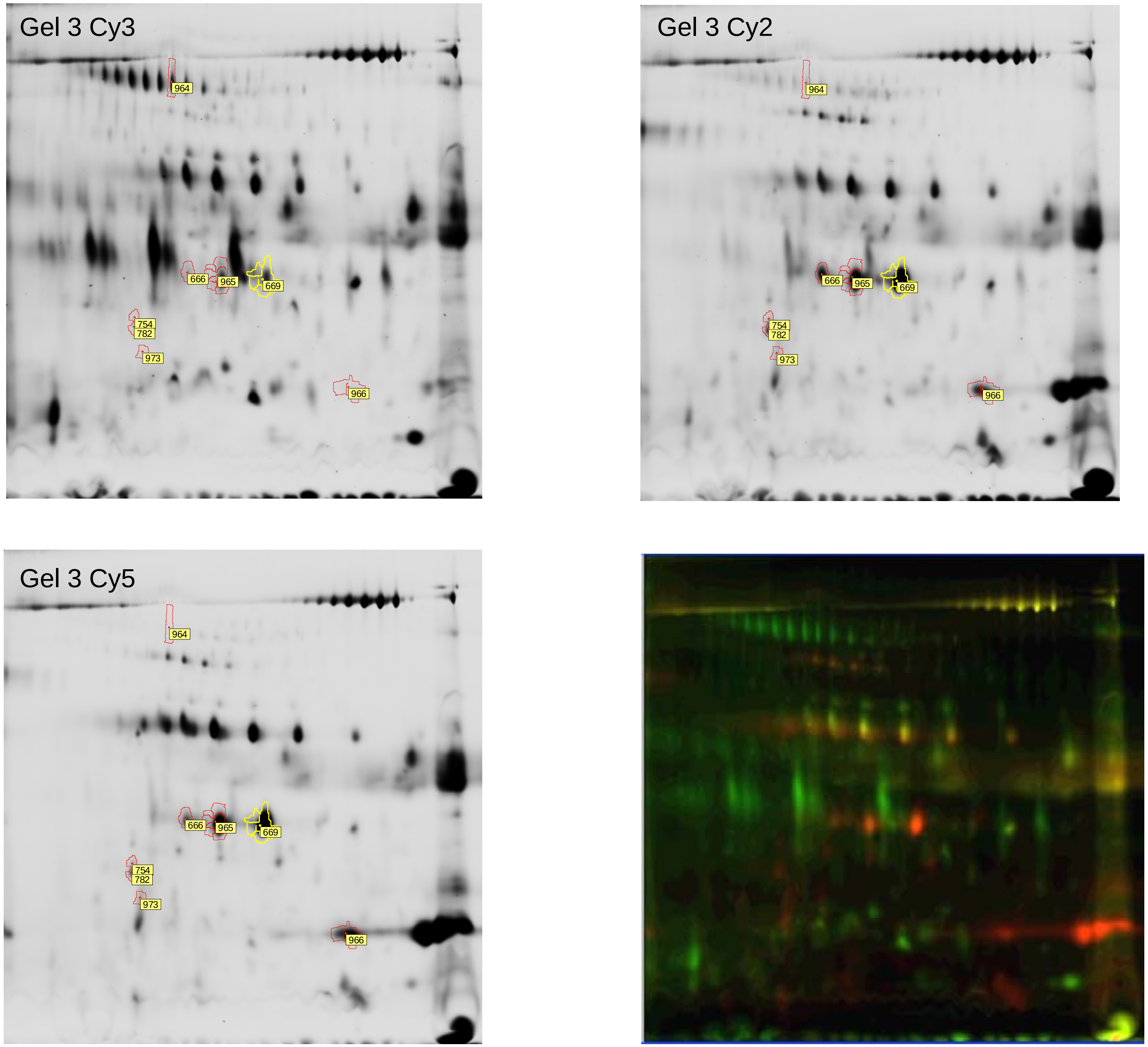

Suppl. Fig. 1. Representative DIGE images (differential spots are marked).

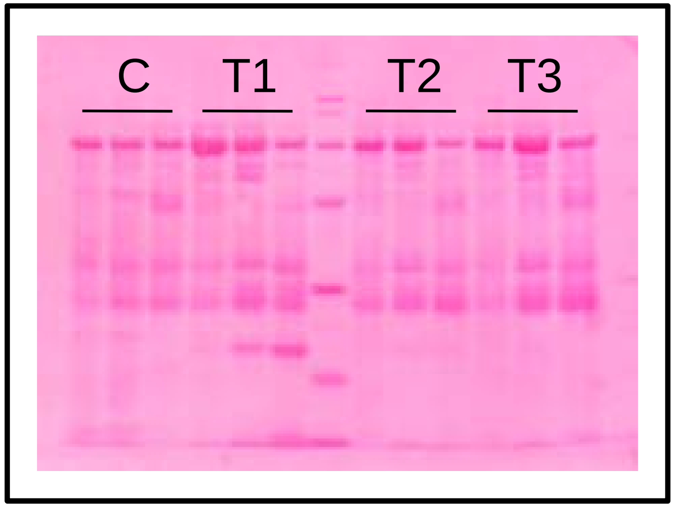

A

Representative Ponceau.

The same amount of total protein was loaded per lane (per sample).

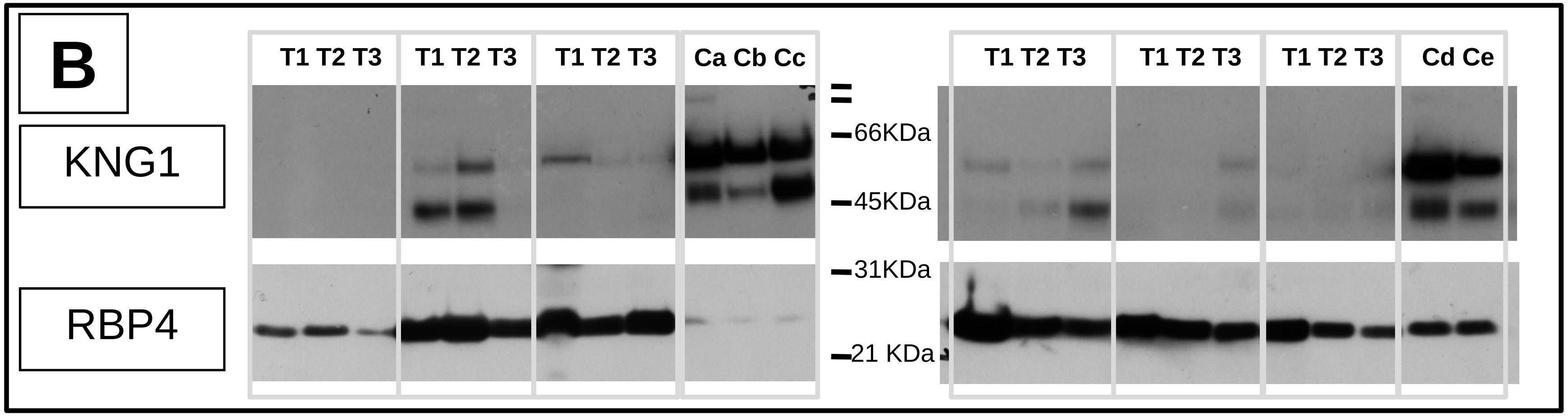

Representative WB images for KNG1 and RBP4.

C: control. T1,T2,T3: patients' samples at the different points of time.

Suppl. Fig. 2. Representative Ponceau image (A) and WB images for KNG1 and RBP4 (B).

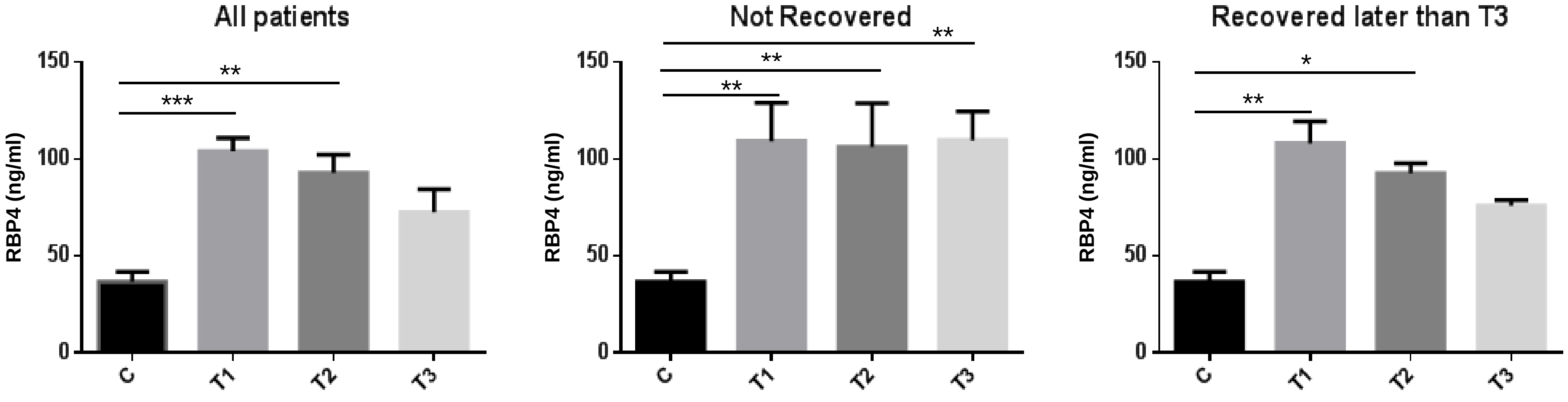

Suppl. Fig. 3. RBP4 response for control subjects (C) and AKI patients at different points of time (T1, T2 and T3) by ELISA assay.

Summary DIGE analysis. Data report

8 spots were found significantly altered and submitted to MALDI-MS/MS analysis for protein identification. In the Table, altered spots and significant variation are shown.

Master No.	Appearance	T1 / C		T2 / C		T3 / C		T2 / T1		T3 / T1		T2 / T3		ANOVA
		T-test	Av. Ratio	T-test	Av. Ratio	T-test	Av. Ratio	T-test	Av. Ratio	T-test	Av. Ratio	T-test	Av. Ratio	
666	30 (30)	0.00038	9.97	0.0098	7.43	0.1	5.42	0.99	-1.34	0.63	-1.84	1	-1.37	0.0018
669	30 (30)	0.00016	30.07	0.0091	23.74	0.1	20.65	0.99	-1.27	0.63	-1.46	1	-1.15	0.00085
754	27 (30)	0.00077	19.06	0.0091	15.69	0.1	9.93	0.99	-1.21	0.63	-1.92	1	-1.58	0.00085
782	30 (30)	0.0014	13.72	0.019	10.77	0.1	9.62	0.99	-1.27	0.63	-1.43	1	-1.12	0.0014
964	27 (30)	0.0013	-9.13	0.0091	-5.57	0.074	-5.76	0.99	1.64	0.63	1.58	1	-1.03	0.00041
965	30 (30)	0.00038	13.64	0.0091	11.51	0.11	9.59	0.99	-1.19	0.63	-1.42	1	-1.2	0.002
966	30 (30)	0.00016	32.9	0.0091	25.35	0.074	13.39	0.99	-1.3	0.63	-2.46	1	-1.89	0.00041
973	24 (30)	0.00077	25.81	0.0091	18.47	0.1	9.6	0.99	-1.4	0.63	-2.69	1	-1.92	0.00041

Master No.: spot code

Appearance: number of images (among the 30) in which the spot is quantifiable

Mass spectrometry data revealed proteins identification in all spots, except 666 and 973:

-669: Basement membrane-specific heparan sulphate proteoglycan core protein (P98160)

-754: Retinol binding protein4 (P02753)

-782: Retinol-binding protein 4, Ig kappa chain V-I region OU (P01606), Serine palmitoyltransferase small subunit A (Q969W0), Ig heavy chain V-III region BUR (P01773), NADH-ubiquinone oxidoreductase chain 4L (P03901) and Reactive oxygen species modulator 1 (P60602).

-964: Kininogen-1 (P01042)

-965: Retinol-binding protein 4 (P02753), Putative uncharacterized protein C1orf196 (B1AJZ1), Ig kappa chain V-I region OU (P01606), Ig kappa chain V-II region MIL (P01616), Reactive oxygen species modulator 1 (P60602), HERV-K_10p14 provirus Rec protein (P61578), Nucleoside diphosphate kinase (O00746), mitochondrial and Ig heavy chain V-III region BUR (P01773).

-966: Polyubiquitin-B (P0CG47), Ubiquitin-60S (P62987), Ubiquitin-40S (P62979), Polyubiquitin-C (P0CG48).

Detail information on MALDI-MS/MS proteins indentification follows for RBP4 and KNG1.

Mascot Search Results

Protein View

Match to: **P02753** Score: **62** Expect: **0.012**
Retinol-binding protein 4 OS=Homo sapiens GN=RBP4 PE=1 SV=3

Nominal mass (M_r): **23337**; Calculated pI value: **5.76**
NCBI BLAST search of P02753 against nr
Unformatted sequence string for pasting into other applications

Taxonomy: Homo sapiens

Fixed modifications: Carbamidomethyl (C)
Variable modifications: Oxidation (M)
No enzyme cleavage specificity
Sequence Coverage: 94%

Matched peptides shown in **Bold Red**

```
  1 MKWVWALLLL AALGSGRAER DCRVSSFRVK ENFDKARFSG TWYAMAKKDP
 51 EGLFLQDNIV AEFSVDETGQ MSATAKGRVR LLNNWDVCAD MVGTFTDTED
101 PAKFKMKYWG VASFLQKGND DHWIVDTDYD TYAVQYSCRL LNLDGTCADS
151 YSFVFSRDPN GLPPEAQKIV RQRQEELCLA RQYRLIVHNG YCDGRSERNL
201 L
```

Sort Peptides By ◉ Residue Number ○ Increasing Mass ○ Decreasing Mass

Start - End	Observed	Mr(expt)	Mr(calc)	ppm	Miss	Sequence
1 - 7	949.5016	948.4943	948.4891	5	0	-.MKWVWAL.L Oxidation (M) (No match)
3 - 9	900.4811	899.4738	899.5269	-59	0	K.WVWALLL.L (No match)
3 - 17	1626.0590	1625.0517	1624.9453	65	0	K.WVWALLLLAALGSGR.A (No match)
5 - 14	1040.5811	1039.5738	1039.6430	-67	0	V.WALLLLAALG.S (No match)
17 - 32	2009.0259	2008.0186	2008.0021	8	0	G.RAERDCRVSSFRVKEN.F (No match)
24 - 35	1455.8405	1454.8332	1454.7518	56	0	R.VSSFRVKENFDK.A (No match)
25 - 32	966.5125	965.5052	965.4930	13	0	V.SSFRVKEN.F (No match)
26 - 32	879.4815	878.4742	878.4610	15	0	S.SFRVKEN.F (No match)
30 - 36	851.4745	850.4672	850.4185	57	0	V.KENFDKA.R (No match)
32 - 46	1780.9094	1779.9021	1779.8039	55	0	E.NFDKARFSGTWYAMA.K Oxidation (M) (No match)

33 - 39	870.4823	869.4750	869.4395	41	0
N.FDKARFS.G (No match)					
42 - 48	897.4965	896.4892	896.4578	35	0
T.WYAMAKK.D (No match)					
42 - 48	913.5018	912.4945	912.4527	46	0
T.WYAMAKK.D Oxidation (M) (No match)					
46 - 53	857.4810	856.4737	856.4654	10	0
M.AKKDPEGL.F (No match)					
54 - 61	919.4905	918.4832	918.4811	2	0
L.FLQDNIVA.E (No match)					
57 - 73	1828.8070	1827.7997	1827.7833	9	0
Q.DNIVAEFSVDETGQMSA.T Oxidation (M) (No match)					
67 - 76	1023.5563	1022.5490	1022.4702	77	0
D.ETGQMSATAK.G (No match)					
68 - 77	951.4855	950.4782	950.4491	31	0
E.TGQMSATAKG.R (No match)					
69 - 78	1006.5499	1005.5426	1005.5025	40	0
T.GQMSATAKGR.V (No match)					
73 - 80	858.4791	857.4718	857.5195	-56	0
S.ATAKGRVR.L (No match)					
76 - 91	1962.9795	1961.9722	1961.9564	8	0
A.KGRVRLLNNWDVCADM.V Oxidation (M) (No match)					
78 - 96	2283.1328	2282.1255	2282.0936	14	0
G.RVRLLNNWDVCADMVGTFT.D Oxidation (M) (No match)					
80 - 86	930.5290	929.5217	929.4719	54	0
V.RLLNNWD.V (No match)					
100 - 107	980.5288	979.5215	979.5161	6	0
E.DPAKFKMK.Y Oxidation (M) (No match)					
101 - 107	865.4801	864.4728	864.4891	-19	0
D.PAKFKMK.Y Oxidation (M) (No match)					
101 - 108	1028.5676	1027.5603	1027.5524	8	0
D.PAKFKMKY.W Oxidation (M) (No match)					
102 - 108	915.5029	914.4956	914.5048	-10	0
P.AKFKMKY.W (No match)					
103 - 108	860.4777	859.4704	859.4626	9	0
A.KFKMKY.W Oxidation (M) (No match)					
104 - 109	902.4962	901.4889	901.4520	41	0
K.FKMKYW.G (No match)					
105 - 111	911.4979	910.4906	910.4735	19	0
F.KMKYWGV.A (No match)					
111 - 119	963.5157	962.5084	962.5185	-10	0
G.VASFLQKGN.D (No match)					
118 - 132	1828.8070	1827.7997	1827.7224	42	0
K.GNDDHWIVDTDYDTY.A (Ions score 63)					
134 - 140	925.5018	924.4945	924.4487	50	0
A.VQYSCRL.L (No match)					
137 - 143	875.4949	874.4876	874.4695	21	0
Y.SCRLLNL.D (No match)					
140 - 148	976.4985	975.4912	975.4695	22	0
R.LLNLDGTCA.D (No match)					
153 - 159	867.4841	866.4768	866.4287	56	0
S.FVFSRDP.N (No match)					
154 - 161	891.4908	890.4835	890.4246	66	0
F.VFSRDPNG.L (No match)					
158 - 166	909.4955	908.4882	908.4239	71	0
R.DPNGLPPEA.Q (No match)					
163 - 170	881.4819	880.4746	880.5018	-31	0
L.PPEAQKIV.R (No match)					
170 - 176	944.4992	943.4919	943.4835	9	0
I.VRQRQEE.L (No match)					

Start - End	Observed	Mr(expt)	Mr(calc)	ppm	Miss	Sequence
176 - 183	1052.5734	1051.5661	1051.5120	51	0	E.ELCLARQY.R (No match)
182 - 189	1042.5443	1041.5370	1041.5719	-34	0	R.QYRLIVHN.G (No match)
193 - 200	946.5038	945.4965	945.4628	36	0	C.DGRSERNL.L (No match)

Mascot Search Results

Protein View

Match to: **P01042** Score: **82** Expect: **0.00012**
Kininogen-1 OS=Homo sapiens GN=KNG1 PE=1 SV=2

Nominal mass (M_r): **72996**; Calculated pI value: **6.34**
NCBI BLAST search of P01042 against nr
Unformatted sequence string for pasting into other applications

Taxonomy: Homo sapiens

Fixed modifications: Carbamidomethyl (C)
Variable modifications: Oxidation (M)
Cleavage by Trypsin: cuts C-term side of KR unless next residue is P
Sequence Coverage: **12%**

Matched peptides shown in **Bold Red**

1 MKLITILFLC SRLLLSLTQE SQSEEIDCND KDLFKAVDAA LKKYNSQNQS
51 NNQFVLYRIT EATK**TVGSDT FYSFK**YEIKE GDCPVQSGKT WQDCEYKDAA
101 K**AATGECTAT VGK**RSSTKFS VATQTCQITP AEGPVVTAQY DCLGCVHPIS
151 TQSPDLEPIL RHGIQYFNNN TQHSSLFMLN EVKRAQR**QVV AGLNFR**ITYS
201 IVQTNCSK**EN FLFLTPDCK**S LWNGDTGECT DNAYIDIQLR IASFSQNCDI
251 YPGKDFVQPP TKICVGCPRD IPTNSPELEE TLTHTITKLN AENNATFYFK
301 IDNVKKARVQ VVAGKK**YFID FVAR**ETTCSK ESNEELTESC ETKK**LGQSLD**
351 **CNAEVYVVPW EKK**IYPTVNC QPLGMISLMK **RPPGFSPFR**S SRIGEIKEET
401 TVSPPHTSMA PAQDEERDSG KEQGHTRRHD WGHEKQRKHN LGHGHKHERD
451 QGHGHQRGHG LGHGHEQQHG LGHGHKFKLD DDLEHQGGHV LDHGHKHKHG
501 HGHGKHKNKG KKNGKHNGWK TEHLASSSED STTPSAQTQE KTEGPTPIPS
551 LAKPGVTVTF SDFQDSDLIA TMMPPISPAP IQSDDDWIPD IQIDPNGLSF
601 NPISDFPDTT SPKCPGRPWK SVSEINPTTQ MKESYYFDLT DGLS

Show predicted peptides also

Sort Peptides By ◉ Residue Number ○ Increasing Mass ○ Decreasing Mass

Start - End	Observed	Mr(expt)	Mr(calc)	ppm	Miss	Sequence
65 - 75	1251.6255	1250.6182	1250.5819	29	0	K.TVGSDTFYSFK.Y (No match)
102 - 113	1165.6155	1164.6082	1164.5445	55	0	K.AATGECTATVGK.R (No match)
188 - 196	1003.6064	1002.5991	1002.5611	38	0	R.QVVAGLNFR.I (No match)

209 - 219	1383.6929	1382.6856	1382.6540	23	0
K.ENFLFLTPDCK.S (Ions score 37)					
317 - 324	1030.5754	1029.5681	1029.5284	39	0
K.YFIDFVAR.E (No match)					
317 - 324	1030.5754	1029.5681	1029.5284	39	0
K.YFIDFVAR.E (Ions score 21)					
345 - 363	2235.2185	2234.2112	2234.1041	48	1
K.LGQSLDCNAEVYVVPWEKK.I (No match)					
381 - 389	1060.5465	1059.5392	1059.5614	-21	0
K.RPPGFSPFR.S (No match)					
381 - 389	1060.5465	1059.5392	1059.5614	-21	0
K.RPPGFSPFR.S (No match)					

SRM analysis conditions in a QQQ-LC/MS/MS.

Protein name	*Precursor mass(charge)*	*Fragment mass*	*Peptide sequence*	*Collision energy*
RBP4	813.48(+2)	970.60(+1)<br>857.52(+1)	WVWALLLLAALGSGR	25.9
KNG1	713.70(+3)	956.0(+2)<br>856.9(+2)<br>637.7(+3)	DIPTNSPELEETLTHTITK	16.6
